# Refining an Asynchronous Telerehabilitation Platform for Speech-Language Pathology: Engaging End-Users in the Process

**DOI:** 10.3389/fnhum.2016.00640

**Published:** 2016-12-20

**Authors:** Annie J. Hill, Hugh M. Breslin

**Affiliations:** ^1^Centre of Research Excellence in Telehealth, School of Health and Rehabilitation Sciences, The University of QueenslandBrisbane, QLD, Australia; ^2^BreezeSoftBrisbane, QLD, Australia

**Keywords:** telerehabilitation, telepractice, speech pathology, aphasia, communication disorders, end-user, usability, acceptability

## Abstract

Asynchronous telerehabilitation in which computer-based interventions are remotely monitored and adapted offline is an emerging service delivery model in the rehabilitation of communication disorders. The asynchronous nature of this model may hold a benefit over its synchronous counterpart by eliminating scheduling issues and thus improving efficiency in a healthcare landscape of constrained resource allocation. The design of asynchronous telerehabilitation platforms should therefore ensure efficiency and flexibility. The authors have been engaged in a program of research to develop and evaluate an asynchronous telerehabilitation platform for use in speech-language pathology. eSALT is a novel asynchronous telerehabilitation platform in which clinicians design and individualize therapy tasks for transfer to a client's mobile device. An inbuilt telerehabilitation module allows for remote monitoring and updating of tasks. This paper introduces eSALT and reports outcomes from an usability study that considered the needs of two end-user groups, people with aphasia and clinicians, in the on-going refinement of eSALT. In the study participants with aphasia were paired with clinicians who used eSALT to design and customize therapy tasks. After training on the mobile device the participants engaged in therapy at home for a period of 3 weeks, while clinicians remotely monitored and updated tasks. Following the home trial, participants, and clinicians engaged in semi-structured interviews and completed surveys on the usability of eSALT and their satisfaction with the platform. Content analysis of data involving five participants and three clinicians revealed a number of usability themes including ease of use, user support, satisfaction, limitations, and potential improvements. These findings were translated into a number of refinements of the eSALT platform including the development of a client interface for use on the Apple iPad®, greater variety in feedback options to both the participant and clinician, automatic transfer of results to the clinician, and expansion of the task template list. This research highlights the importance of including end-users in the process of technology refinement, in order to ensure effective and efficient use of the technology. Future directions for research are discussed including clinical trials in which the effectiveness of and adherence to intervention protocols using asynchronous telerehabilitation are examined.

## Introduction

Aphasia is an acquired communication disorder that impedes to varying degrees an individual's comprehension and expression of language, be it through their ability to read, understand spoken language, write, speak, and/or gesture (Code, 2010). Aphasia is most commonly caused by stroke with ~30–40% of acute stroke cases presenting with aphasia (Pedersen et al., 2004; Engelter et al., 2006), and up to 61% of those individuals continuing to experience aphasia at 12 months post-stroke (Pedersen et al., 2004). Therefore, aphasia becomes a chronic communication disorder for a significant proportion of people who experience stroke. Fortunately, research has demonstrated that people with aphasia can continue to improve their language skills many years post-stroke when they have access to efficacious treatment regimens (Brady et al., 2012). While a number of studies have suggested that intensive aphasia rehabilitation is most effective (Bhogal et al., 2003; Kelly et al., 2010; Cherney et al., 2011), other research supports more distributed protocols (Dignam et al., 2015). A likely outcome of this debate about rehabilitation intensity is that rehabilitation interventions should be tailored to the individual's needs. However, full customization of aphasia rehabilitation protocols that are delivered in-person are improbable in a healthcare landscape of increasingly restricted resource allocation combined with growing demand. Furthermore, even when such tailored rehabilitation services are available, access by individuals with aphasia may be restricted due to decreased mobility and/or geographical isolation. Fortunately, a number of technology-based solutions, such as computer-based aphasia therapy (CBAT) and telerehabilitation, have been implemented in aphasia rehabilitation to overcome these issues of access, intensity, and limited resources.

While CBAT programs have been used in aphasia rehabilitation for many years (van de Sandt-Koenderman, 2011) only recently has the highest level of evidence supporting the use of CBAT been reported in a systematic review (Zheng et al., 2016). The review concluded that there is evidence of the effectiveness of CBAT for people with chronic aphasia as it found statistically significant improvements in language outcomes after CBAT when compared to no therapy in all but one study and no statistically significant differences in language outcomes when compared to traditional clinician-delivered therapy. Nevertheless, the authors of the review recommended that clinicians carefully consider matching the CBAT program to the needs of each client in terms of feedback, cueing hierarchies and the degree of clinician support provided when clients use CBAT. This recommendation may result in clinicians needing access to a wide range of CBAT programs in order to tailor therapies to this complex and heterogeneous disorder given that most CBAT programs are currently fixed in terms of the types of tasks, feedback and cues available. Zheng et al. (2016) called for further quality and controlled research to be conducted around the use of CBAT in order to determine the key features required in CBAT programs and treatment protocols best suited to various types and severities of aphasia. This call for further research was also made by van de Sandt-Koenderman (2011) who appealed for current CBAT programs to be made more sophisticated and attractive to users by bringing them up-to-date with new technologies such as mobile devices. Additionally, van de Sandt-Koenderman (2011) called for the development of CBAT programs that encompass functional treatment goals and social participation needs of people with aphasia, as well as, expansion into telerehabilitation models in which a clinician guides and supports a client in their rehabilitation using CBAT programs.

Telerehabilitation is another technology-based solution that has been explored for both assessment and treatment of aphasia. In the context of speech-language pathology telerehabilitation (or its synonym telepractice) has been defined by the peak professional body Speech Pathology Australia, as “the application of telecommunications technology to deliver clinical services at a distance by linking clinician to client, caregiver, or any person(s) responsible for delivering care to the client” (SPA Position Statement, 2014). Telerehabilitation may be delivered synchronously (real-time), or asynchronously (delayed), or using a hybrid combination of the two approaches. Much of the aphasia telerehabilitation research to date has focused on synchronous or hybrid approaches (see Systematic Reviews by Hall et al., 2013; Coleman et al., 2015). In contrast, there has been less research into asynchronous models of telerehabilitation for aphasia rehabilitation reported in the literature.

Seminal research into asynchronous telerehabilitation was conducted by Mortley and colleagues who combined the use of a CBAT program, StepByStep, with an asynchronous telerehabilitation approach to deliver aphasia rehabilitation directly into the individual's home (Wade et al., 2003; Mortley et al., 2004). The CBAT program, StepByStep had been designed for independent use by people with aphasia and was accessed on a personal computer with a Windows operating system. A range of tasks were available such as written and spoken word to picture matching, semantic association tasks, as well as, naming, reading, and spelling tasks. An important feature of the StepByStep program was that it allowed for the input of personal and meaningful stimuli. In this way the program aligned with the neurorehabilitation principle of saliency, which is a significant factor in language recovery (Pulvermüller and Berthier, 2008; Raymer et al., 2008). The telerehabilitation approach allowed clinicians to remotely monitor progress, provide feedback and update therapy tasks, resulting in an improved ratio of therapy to therapist time when compared with other computer-based aphasia therapy programs (Mortley et al., 2004). Clinical results were encouraging in that all seven participants demonstrated consistent and intensive use of the program, resulting in significant improvements in word retrieval (Mortley et al., 2004). Furthermore, the participants reported experiencing increased confidence and participation in communication. The participants credited the asynchronous telerehabilitation model with improving their access to therapy, increasing the intensity of therapy and increasing their autonomy (Wade et al., 2003). This research highlighted the importance of providing people with aphasia with salient therapy tasks and demonstrated that people with aphasia appreciated having access to clinician monitored therapy which they could engage in autonomously. However, the uptake of the Windows-hosted StepByStep program has perhaps been impeded by recent advances in technology toward mobile touch devices (iPad®, tablet PC, smartphones). As interest in mobile devices continues to grow current programs may need to be converted or new programs developed. Furthermore, research suggests that tomorrow's elders with disabilities will demand mobile access to services as they continue to participate in a lifestyle which includes travel and other activities outside the home (Morris et al., 2010).

In response to this burgeoning use of mobile devices in the community, Brandenburg and colleagues conducted an integrated review of mobile computing and aphasia which examined issues of accessibility for people with aphasia and the potential uses of mobile computing with this population (Brandenburg et al., 2013). The issue of accessibility (also called usability) in CBAT programs is critical to the successful uptake of these programs. Usability has been defined by the International Standards Organization (ISO) as “the extent to which a product can be used by specified users to achieve specified goals with effectiveness, efficiency, and satisfaction in a specified context of use” (ISO 9241-11, 1998). Another important concept in the development of software and new service delivery models such as telerehabilitation is acceptability. Acceptability is a judgment by the user of the software or service as to whether their needs are sufficiently met. As discussed by Brandenburg et al. (2013) a number of features have been identified as improving the usability of mobile technology for people with aphasia. However, more research is needed in this area and the use of participatory design methodology, or an end-user design approach as it is known in the software development field, may be one way to achieve improved usability of CBAT programs accessed on mobile devices. Given that mobile devices are becoming the device of choice for many people, including people with aphasia (Brandenburg et al., 2013), it is encouraging that recently developed asynchronous telerehabilitation platforms have made use of this new technology (Kiran et al., 2014; Brandenburg et al., 2015; Des Roches et al., 2015; Choi et al., 2016). Brandenburg et al. (2015) developed an application for use on a smartphone which measures “talk time” in much the same way that a pedometer measures steps. Features of CommFit™ include being able to set a goal for the amount of talking, and data feedback to both the person with aphasia using the app and their clinician. Using key usability design and aphasia friendly text principles for people with aphasia, the prototype application was developed and then trialed with healthy adults to determine the accuracy of the measurement of talking time. Usability of this app with the target population is yet to be published.

The other two recently reported asynchronous telerehabilitation systems are more complex in that the programs aim to provide language therapy across a number of domains. Kiran et al. (2014) and Des Roches et al. (2015) reported the use of an iPad®-based software program, Constant Therapy, to deliver a range of impairment-level language and cognitive tasks to participants with aphasia. The Constant Therapy program also allowed clinicians to monitor therapy progress remotely. Kiran et al. (2014) demonstrated the feasibility of the iPad®-based software platform to deliver therapy tasks to four participants. Tasks were assigned from the Constant Therapy library of approximately 30 evidence-based language and cognitive therapy tasks, each with varying levels of difficulty thereby allowing some degree of individualization of therapy. Following the positive findings of this feasibility testing, Des Roches et al. (2015) investigated the effectiveness of the program in a comparison study where both groups received ten 1-h sessions to complete therapy tasks on the iPad with the support of a clinician and the experimental group supplemented these sessions with home practice on the iPad® between clinic visits. Both the experimental and control groups showed improvements on standardized testing, however, the experimental group showed greater improvements than the control group, thus demonstrating the benefits of increased practice time using Constant Therapy. While the effectiveness of this CBAT program is promising, the researchers noted only 68.7% compliance with the prescribed 6 h of home practice. It would be interesting to determine whether usability issues impacted compliance and client satisfaction with this iPad®-based therapy approach.

The asynchronous telerehabilitation platform described by Choi et al. (2016), iAphasia, was also designed for use on an Apple iPad®. The iAphasia program consisted of therapy tasks across the six language domains of auditory comprehension, reading comprehension, repetition, naming, writing, and verbal fluency. Six levels of difficulty were available for each domain and arranged hierarchically. Automatic feedback and progress graphs were available to the participant with aphasia. Clinicians accessed client data from iAphasia stored on a database through a Web portal. Results from a feasibility study using iAphasia with eight participants were promising with significant improvement on standardized assessment after the 4 week trial and maintenance of these outcomes at follow-up 4 weeks later. Furthermore, a strong and positive correlation was found between usage time and improvement in standardized assessment scores regardless of aphasia severity at baseline. Interestingly, participants with mild aphasia had low usage time which the authors concluded was due to the program not being “attractive” to these participants. Regrettably, “attractive” was not defined and so it is unclear whether this was due to issues of usability or acceptability of the program, or its limited capacity to meet the participant's goals and needs due to the restriction of six domains and six difficulty levels.

Thus, while the asynchronous telerehabilitation platforms that make use of modern mobile devices hold great promise for improving access to therapy, there are some limitations in their application. In particular the fixed therapy task types and difficulty levels within some of the programs (e.g., Constant Therapy and iAphasia) may restrict their use to people with specific aphasia profiles. The inability to import client images and/or local audio content to increase the saliency of tasks is also perhaps a limitation of these newer programs. Interestingly, the older program StepByStep had this capacity to import images, but its utilization of Windows operating system restricts somewhat its accessibility on mobile devices. Indeed to the best of the authors' knowledge, none of the asynchronous telerehabilitation programs for aphasia published to date have the capacity to provide complete customization of therapy tasks such that the programs can be used with a wide variety of aphasia types and severities to meet the goals of the user. Furthermore, although the asynchronous telerehabilitation studies reviewed here have implemented aphasia friendly principles into the design of their user interfaces, none have scientifically examined the usability of their programs with people with aphasia. Therefore, there is a need to develop an asynchronous telerehabilitation platform that has the capacity to be used across a range of clients of varying severity and aphasia types, and which is flexible in terms of the types of tasks available for use with clients or easily adaptable. It is important that the platform uses high quality stimuli that are culturally appropriate and ideally would allow for the import of client images and local audio content to increase saliency. Furthermore, usability of the asynchronous telerehabilitation platform should be considered throughout the development period.

Usability issues are a major barrier to the uptake of technology for people with disabilities (Simpson et al., 2010). For people with aphasia it is not just the communication disorder that presents challenges to technology usability, but also the sensory, physical, and cognitive impairments that often accompany aphasia (Brandenburg et al., 2013). One way to potentially ensure adequate usability of CBAT programs, either used in isolation or as part of an asynchronous telerehabilitation system, is to include end-users in the design and development of the programs and to assess usability with the target populations. Considered best practice within software development, the end-user design process aims to inform the development of a product by considering the requirements of the user at all stages of the design process (Pavelin et al., 2012). However, the methodology of the end-user design approach relies heavily on communication skills and so the inclusion of people with aphasia as participants in the end-user design process may be particularly challenging (Galliers et al., 2012). Nevertheless, a number of studies have implemented aspects of the end-user design approach in order to enhance the usability of CBAT programs for people with aphasia (Boyd-Graber et al., 2006; Allen et al., 2008; Koppenol et al., 2010; Galliers et al., 2012; Simic et al., 2016). The study which most comprehensively included end-users in the design approach was that by Galliers et al. (2012) which aimed to develop a gesture therapy tool for people with aphasia. Five people with aphasia acted as consultants in a series of participatory workshops with developers. Furthermore, the Galliers et al. (2012) study provided researchers and developers alike with useful strategies in how best to promote the participation of people with aphasia in this end-user design approach. The Simic et al. (2016) study included both end-user groups in their usability study involving a synchronous telepractice program, PhonoCom. In keeping with the end-user design process, this paper explores the development of a novel asynchronous telerehabilitation platform, called eSALT (eSALT: Enabling Speech and Language Therapy that is effective, electronic, and everywhere) and reports data from a usability study with two end-user groups and discusses how this data was used to further refine eSALT.

### Aim of the current study

The current usability study is part of a broader program of research which aims to develop and comprehensively evaluate an asynchronous telerehabilitation platform for use in speech-language pathology (eSALT) which is flexible enough to accommodate the rehabilitation needs of a wide variety of users.

The aim of this usability study was to explore the usability and acceptability of eSALT from the perspective of participants with aphasia and speech-language pathologists.

## Materials and methods

### Initial development of eSALT

eSALT is an implementation of a generic software program developed by the second author, Hugh Breslin. The generic program, AppTailor, allows the user to easily and quickly design and/or adapt applications or “apps” for deployment to mobile devices. The AppTailor system has two parts, a desktop application and a device-based component. The desktop application allows practitioners to design tasks to be performed by their clients on a mobile device. The user interface provides a preview of how the task will appear on the device. The designer can build a layout for each task and specify which illustrated words or phrases will be used in each step of the task. Additionally standard controls such as dropdown boxes, text editor, and switch controls can be dropped onto the layout as well as media elements such as a video player or a voice recorder. The saved designed task serves as a set of “layout instructions” that can be assigned to device users and uploaded to the cloud. The device application downloads and reads the “layout instructions” to determine how the device app should look and behave. The data input captured is uploaded to the cloud. The desktop application can download these results and present them to the practitioner so that they can view results and modify the tasks accordingly to repeat the process.

The authors felt that the entire AppTailor system could be adapted and implemented as an asynchronous telerehabilitation platform for use in speech-language pathology. A period of adaptation of AppTailor into eSALT followed during which the nomenclature was made specific to speech pathology, custom layouts were developed, and the word/picture library and audio cues were created. The result was a working prototype of eSALT (v1.1) which allowed clinicians to develop and customize impairment-based aphasia therapy tasks for transfer to Windows tablet devices from which clients with aphasia could access the tasks.

As an asynchronous telerehabilitation platform, eSALT has both a client interface and a clinician interface and so aspects of end-user design and examination of usability needed to extend to the clinician's use of the platform to monitor progress, provide feedback and update tasks remotely. Hence, a qualitative study with speech-language pathologists (SLPs) experienced in aphasia rehabilitation using CBAT was undertaken to determine SLPs' preferences for a comprehensive CBAT program (Swales et al., 2016). Ten Australian SLPs participated in this study. Five themes emerged from the thematic analysis which detailed preferences across therapy activities in the CBAT program, the stimuli used and cues available, as well as, preferences for accessing progress data and considerations around accessibility and usability (Swales et al., 2016). Importantly, these SLPs confirmed that there was a need for a CBAT program that was more flexible than current programs in the types of therapy tasks available and that there should be a high degree of flexibility in the program for “clinicians to set all task parameters to each client's needs and increase saliency of therapy” through specific targets, stimuli, cues and feedback (Swales et al., 2016, p. 326). The SLPs considered usability of CBAT programs from a number of perspectives other than their own, such as the individual with aphasia, their family and also other health professionals such as allied health assistants who often manage the use of CBAT programs in clinics. Finally, the SLPs desired a program that was accessible across a variety of operating systems and devices and they confirmed the need for platforms that enabled remote monitoring of client progress and remote updating of therapy tasks. Importantly, these findings came from a group of SLPs who had no experience of eSALT (v1.1). At this point it was decided to embark upon a usability study with both end-user groups, in order to determine the usability and acceptability of eSALT (v1.1).

### Study design

This formative usability study was embedded in a feasibility study and used mixed methods to examine the usability and acceptability of eSALT (v1.1) from the perspective of both people with aphasia and SLPs. The feasibility study involved SLPs designing tasks for the participants with aphasia, remotely monitoring therapy progress and updating tasks. Participants with aphasia engaged in the tablet-based therapy tasks for a period of 3 weeks. Ethical approval was granted by the University of Queensland Behavioural and Social Sciences Ethical Review Committee and all participants provided written informed consent prior to participation. The study took place in 2013.

### Participants

Participants with aphasia were recruited via the Communication Research Register, a database of potential research participants hosted by The University of Queensland. All participants had aphasia as a result of a stroke and were native speakers of English. Participants who presented with concomitant severe apraxia of speech, severe motor speech disorder, cognitive impairment or poorly aided vision and hearing were excluded from the study. A total of five individuals diagnosed with aphasia as a result of stroke (4 males and 1 female) were recruited. The presence of aphasia was confirmed through assessment on the Comprehensive Aphasia Test (CAT; Swinburn et al., 2004). The participants ranged in age from 67 to 78 years old (mean = 70.8 years) and were between 3 and 19 years post-stroke (mean = 8 years, 10 months). Participants were not receiving any other therapy at the time of participation in this study. Characteristics likely to affect the participants' ability to use the tablet PC were also collected (see Table 1 for participant details).

**Table 1:** **Demographic information for participants with aphasia**.

**Participant**	**P1**	**P2**	**P3**	**P4**	**P5**
Gender	M	F	M	M	M
Age (years)	78	69	68	67	72
MPO	228	174	57	39	36
Handedness	R	R	R	R	R
Education level	University (Bachelor Degree)	Grade 12	Grade 10	Grade 7	Diploma
CAT modality mean (T-score)	55.88	49.63	62.5	57.13	41.13
Physical weakness	Nil	R) hemiplegia	R) hemiplegia	Nil	R) hemiplegia
Vision Impairment	Reduced vision R) eye, reading glasses	Reading glasses	Glasses	Nil	Reading glasses
Motivation to do therapy^a^	1	2	1	2	1
Experience using a computer^b^	4	3	1	2	1
Experience using a tablet^b^	3	5	2	2	2
Previous computer-based aphasia therapy	No	Yes	No	No	Yes

*MPO, Months post-onset; CAT, Comprehensive Aphasia Test (Swinburn et al., 2004)*.

a *On a 5 point scale, where 1 = highly motivated, and 5 = not at all motivated*.

b *On a 5 point scale, where 1 = very experienced, and 5 = not at all experienced*.

*R, Right*.

Clinician participants were recruited through their clinical affiliation with the CCRE (the Centre for Clinical Research Excellence) Aphasia Rehabilitation hosted at The University of Queensland and had a minimum of 3 years' experience in aphasia management. Three clinicians participated in this usability study. Clinician participants provided written informed consent. Each clinician completed a demographic survey which also asked about experience using computers and tablet devices in aphasia rehabilitation (see Table 2).

**Table 2:** **Demographic information for clinician participants**.

**Participant**	**SLP1**	**SLP2**	**SLP3**
Gender	F	F	F
Qualification	MSpPath	BSpPath, Masters of Community Rehab	BSpPath (Honours class 1)
Years working in aphasia rehabilitation	4	4	11
Proportion of career spent working in aphasia rehabilitation	50%	90–100%	90–100%
Current workplace setting	Private hospital - Inpatient rehabilitation	Private practice Private hospital - Inpatient/outpatient rehabilitation	Private practice Private hospital - Acute
Previous workplace setting	Private hospital - Acute, inpatient/outpatient rehabilitation	Public Hospital - Acute, inpatient/outpatient rehabilitation Private hospital - Acute - Aged Care	Public Hospital - Acute, inpatient/outpatient rehabilitation Private hospital - Acute, inpatient/outpatient rehabilitation Public community health Aged Care
Level of confidence using computers^a^	Very comfortable	Somewhat comfortable	Somewhat comfortable
Proportion of a therapy session spent using computers	>50% of session	25–50% of session	>50% of session
Use of tablet device with clients with aphasia	25–50% of clients	50–75% of clients	50% of clients
Proportion of therapy session using tablet device	>50% of session	>50% of session	>50% of session
Use of telerehabilitation in clinical practice	None	None	None

a *4 options were: I avoid using computers, somewhat uncomfortable; somewhat comfortable and very comfortable*.

### Materials

#### Hardware

The participants were provided with a tablet PC, either Asus Vivo Tab Smart tablets loaded with Windows 8 software or Asus EP121 tablet that used a Windows 7 operating platform. Each participant was provided with a mobile broadband device to allow them to access the Internet for transfer of therapy results and updated tasks. The mobile broadband device and tablet PC were configured to automatically pair to allow this transfer of data to occur. The treating clinicians were provided with a laptop (Asus A55VD-SX145S) to design and customize therapy tasks, monitor participant progress and update therapy tasks.

#### Software

**Figure 1:**
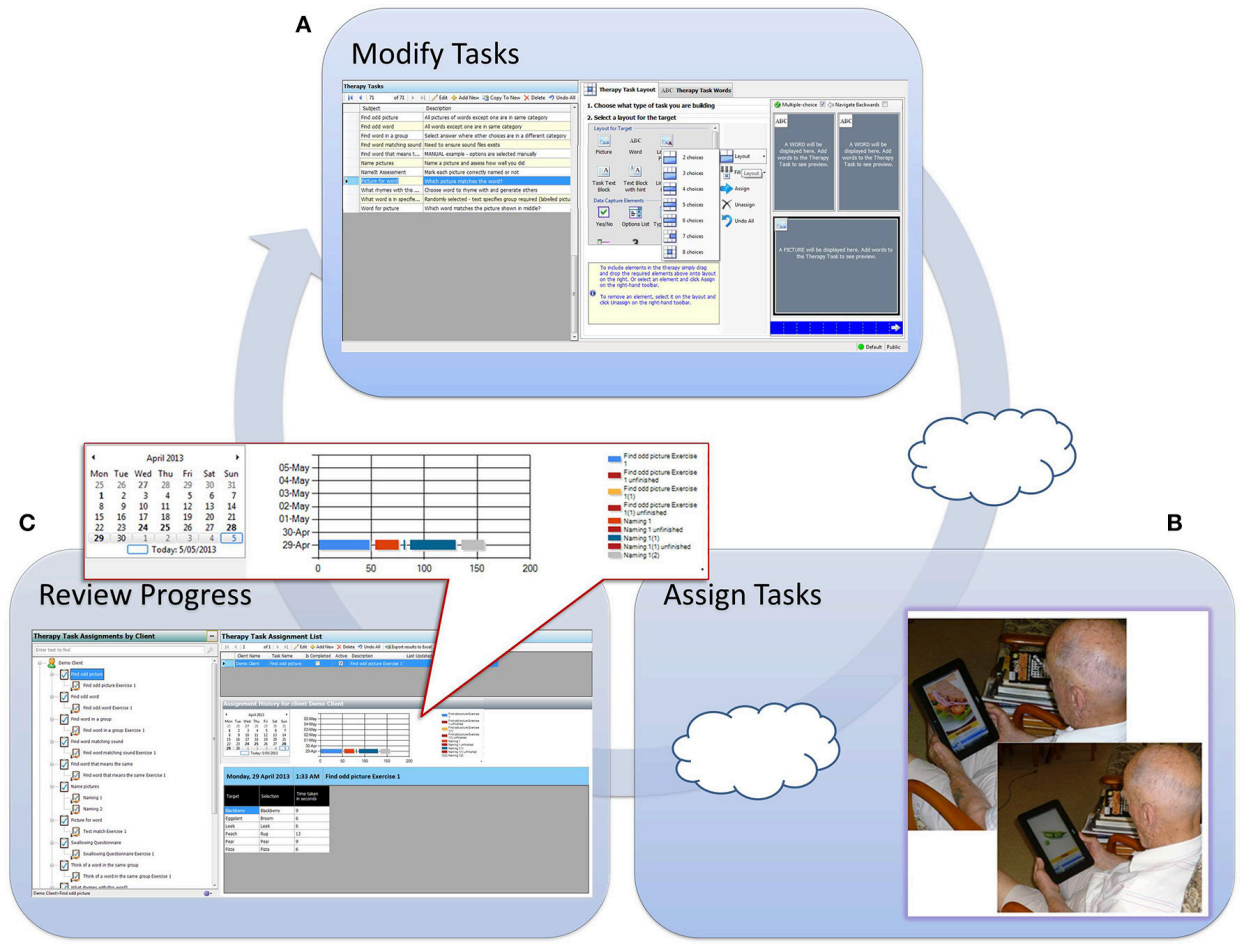
**Depicts process flow when using eSALT**. **(A)** Task design screen from eSALT 2013; **(B)** client using eSALT to access tasks on Windows tablet; **(C)** Remote monitoring screen from eSALT 2013.

The screens from the desktop application of eSALT (v1.1) that the clinicians used to develop and customize therapy tasks and remotely monitor their participant's progress are depicted in (Figures 1A,C). The key features of eSALT (v1.1) included a word/picture library containing over 800 high quality color photographs, a list of 21 task templates (including tasks for word retrieval, semantics, auditory comprehension, reading comprehension), audio and written cues for all words in the library and video cues for ~122 words/pictures, audio capture and playback. These features enabled the clinicians to either develop therapy tasks from scratch or alter the task templates to suit their participants and to easily randomize the distractors in the task. The clinicians also had access to results at a task level and could export all data to Excel for further analysis. All data was de-identified via a coded reference in the application and stored on a secure server in a standard format.

The tablet application of eSALT (v1.1) that the participants with aphasia used interpreted the task designed by the clinician, displaying selected words, pictures, cues etc. The application also provided immediate feedback to the participant on therapy tasks requiring a touch response (e.g., word to picture matching task), allowed participant's to record their verbal expression and play back to themselves. The participant did not need a headset microphone to record their responses; they could record free-field. Audio recordings were only available locally; they were not uploaded to the clinician. Where audio recording was used in a task, the participant would then have to make a judgment about the accuracy of their performance (comparing it to the audio cue) and this was the data transferred to the clinician. Synchronization between the tablet application and the server was manually controlled. (See Figure 1B).

### Procedure

#### Pre-treatment testing

The presence and nature of each participant's aphasia was confirmed by assessment using the CAT (Swinburn et al., 2004) administered by the treating clinician. The results of this assessment and informal conversations with the participant with aphasia were used by the clinicians to develop therapy tasks using eSALT (v1.1).

#### Training

The clinician participants received individual or paired training of up to 2 h on the use of the eSALT (v1.1) software for the development of therapy tasks. Additionally, clinicians were provided with a manual detailing step-by-step procedures for task development, monitoring and upload. Clinicians had access to the lead researcher (AH) throughout the trial to troubleshoot any technical issues.

The participants with aphasia (and carers where applicable) received 1 h of training from the treating clinician in how to use the tablet and complete the tasks assigned to them. To check understanding of procedures to complete tasks, the participants then independently used the tablet PC to complete therapy tasks in the clinic room. The therapy results were transferred to the clinician and at least one therapy task was updated during this session to ensure the telerehabilitation cycle was functional. Participants with aphasia were provided with a user manual containing instructions on the operation of the tablet, mobile broadband device, and eSALT program for reference at home. This user manual was formatted according to aphasia-friendly guidelines outlined by Rose et al. (2011). Participants with aphasia were also supplied with the contact details of the lead researcher (AH) and their clinician and were encouraged to make contact if technical issues arose during the trial period.

#### Treatment

Participants with aphasia practiced their personalized therapy tasks at home over a 3-week period. Performance data were transferred via the Internet from the device to the treating clinician who accessed this data via the laptop. Therapy tasks were updated by the clinician according to their clinical judgment of participant progress and were remotely transferred via the Internet. Participants with aphasia were instructed to practice therapy tasks on the tablet PC daily, for a minimum of half an hour with no upper limit.

### Outcome measures

#### Observational checklist

An independent researcher visited each participant at home once in the first week of the trial. During this visit the participant's ability to use the device to complete therapy tasks and transfer the data was assessed using an observational task checklist which rated the participant's level of independence on a range of tasks related to the operation of both the tablet PC and eSALT (v1.1) program. The checklist used a 5-point scale where 1 = not at all independent, 2 = minimally independent, 3 = moderately independent, 4 = mostly independent and 5 = totally independent. Field notes were also recorded in order to provide more detailed information about each participant's ability to independently use and operate the tablet PC and eSALT (v1.1) program. If required, the researcher also provided further training regarding the use of the tablet PC, eSALT program, or technical difficulties.

Clinicians were observed and rated in terms of their independent use of eSALT (v1.1) at the training session for the participant (i.e., after they had been trained and had used eSALT to develop tasks for their participants). The clinician observation checklist evaluated usability across task design and development and assigning tasks to clients. A four-point scale was used where 1 = aware of feature; 2 = demonstrated with personal assistance; 3 = demonstrated with manual; 4 = demonstrated independently.

#### Post-treatment interview with participants

Following the 3-week home-based trial, a semi-structured interview was conducted by an independent researcher with each participant (both participants with aphasia and clinician participants). The interviews lasted between 50–70 min and participants were provided with the option to terminate the interview at any point, however none wished to do so. With consent, the interviews were audio recorded for subsequent transcription and analysis. As recommended by Galliers et al. (2012), the software program and tablet PC were made available to participants with aphasia during the interview in order to provide concrete visual aids for participants and assist with recall of specific aspects of the tablet PC and eSALT (v1.1). Likewise, the laptop and eSALT (v1.1) program were available to the clinician participants during the interviews. The interviews with participants with aphasia were conducted in line with principles and techniques outlined by Patton (1987), which have been used in similar studies investigating the views of people with aphasia (Wade et al., 2003). Topic guides were developed in order to explore participants' experiences and perceptions regarding the usability of the tablet PC and eSALT (v1.1) program and their satisfaction with the quality of service provided. The topic guides were developed to incorporate several key topics (e.g., usability, remote monitoring, and satisfaction) related to the overall design and purpose of eSALT in order to collect specific information relating to the aims of the study. Probing questions were included in order to explore participants' opinions in depth. However, participants were also encouraged to raise novel issues of particular relevance to them. Although the exact wording and order of the questions differed between interviews, the interviewer ensured that open questions were posed and that questions were worded carefully in order to avoid leading participants toward a certain response. If the interviewer felt that comprehension difficulties were affecting the participants with aphasias' ability to respond to questions, clarification was sought by posing closed questions, i.e., “So you found it easy to do by yourself?” The interviewer also encouraged participants to elaborate on their responses when only general statements were given.

For P2, who presented with concomitant moderate apraxia of speech, and P5, who presented with significant expressive difficulties, the interviews were structured differently in order to facilitate their independent involvement in the interview process. This was done through the use of word and picture cards which participants were able to sort in order to provide detailed responses to questions asked by the interviewer. This method has been reported in the literature as an appropriate way to support the communication of people with aphasia (Kagan, 1998; Palmer et al., 2013).

#### Satisfaction survey

Clinicians also completed a 52-item satisfaction survey to evaluate the clinician's satisfaction with using eSALT (v1.1) and their capacity to deliver appropriate therapy to their participants with aphasia. The survey consisted of 30 five-point ratings scales (1 = Strongly disagree to 5 = Strongly agree), seven multi-select questions, three open-ended questions and 12 open response boxes which opened depending on previous responses.

### Data analysis

For the participants with aphasia, the independent researcher's field notes from the home visits were explored thematically alongside their interview data and results from the observational skills assessment were analyzed using descriptive statistics. The results from the clinician observation checklist and satisfaction survey were analyzed descriptively. Comments, responses to open-ended questions and the multiple-select questions in the survey were analyzed qualitatively alongside the interview data. All responses obtained from the post-treatment semi-structured interviews with both participant groups were transcribed and analyzed separately using content analysis methodology (Graneheim and Lundman, 2004). The qualitative analysis of the interviews was completed by one researcher and then cross-checked by an independent coder using line-by-line analysis and any discrepancies were discussed until agreement was reached about the coding system. This was done in order to enhance the reliability of the outcomes.

## Results

### Participants with aphasia

#### Observational checklist

**Figure 2:**
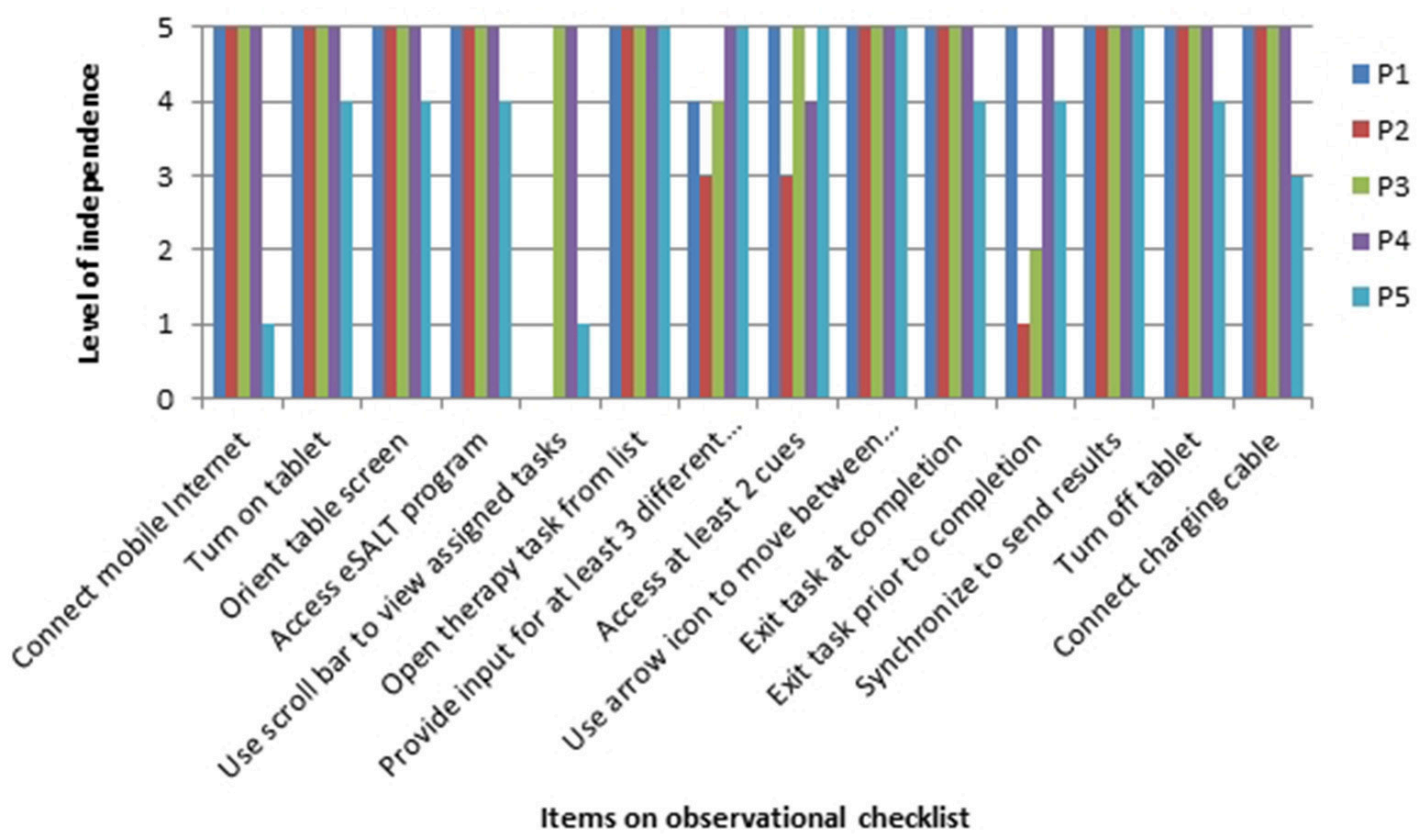
**Ratings of participants with aphasias' level of independence when using eSALT**.

The results of the observational skills assessment provided information regarding each participant's ability to independently engage in the therapy sessions using eSALT (v1.1) on the tablet PC. Figure 2 displays each participant's rating on each task. All participants were completely or mostly independent in 8 of the 14 skills. Areas of difficulty that were identified for some of the participants included using a range of response modes (e.g., keyboard entry, check box selection); accessing a range of cues when completing therapy tasks (e.g., an audio cue, written word cue, and video cue); and exiting therapy tasks prior to completion. Use of the scroll bar to view assigned therapy tasks in the menu was not observed for P1or P2 as they did not have sufficient tasks assigned to necessitate the use of a scroll bar, however P5 could not use the scroll bar independently.

#### Semi-structured interviews

Qualitative analysis of the interview data revealed five main categories: (1) usability; (2) support to use eSALT; (3) telerehabilitation; (4) self-management; and (5) satisfaction. These categories directly addressed the usability (categories one, two, and three) and acceptability (categories four and five) of the system and identified important factors that contributed to the system's usability and participants' overall satisfaction.

##### Category 1—usability

Four sub-categories were identified with the category of usability: Usability of the tablet PC, usability of the eSALT program, the ease of learning to use the program and personal factors that affected usability, as summarized in Supplementary Table 1. Field notes recorded by the research assistant during the home visits provided additional information regarding participants' abilities to successfully use the tablet PC and eSALT program.

**Figure 3:**
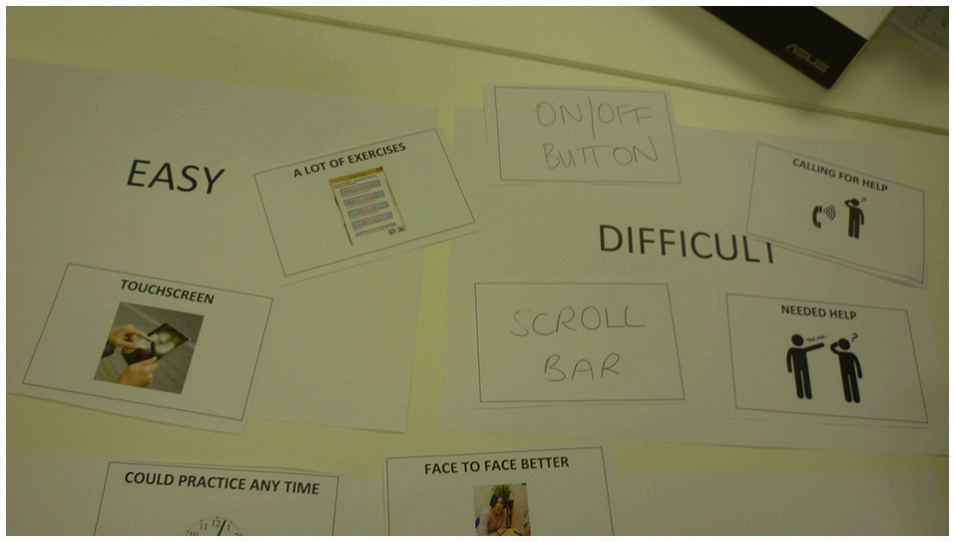
**P2's response to interview questions about ease of use of eSALT**.

All participants felt that they were able to use the eSALT (v1.1) program successfully. Three participants reported that they were able to complete their personalized electronic therapy activities completely independently. The picture stimuli used in eSALT were identified as a feature that enhanced the usability of the system. However, participants also identified features that negatively affected the usability of the eSALT (v1.1) program, such as the scroll bar on the menu which some participants found difficult to manipulate (see Figure 3 for P2's results). Likewise, the participants had mixed views on how easy it was to use the tablet PC. Those who believed the tablet PC was difficult to use reported struggling with the on-screen keyboard and the tablet PC's responsiveness to touch. These difficulties were also observed by the research assistant during the home visit, such as participants tapping the touch screen with too much force. Other participants noted features that positively influenced the tablet PC's usability, such as the size of the screen.

With respect to the ease of learning to use the system, most participants felt that they required training and practice in order to learn how to use it. However, participants felt that their ability to use the program improved over the course of the trial. Participants also commented on personal factors that influenced usability. Two of the participants felt that their limited previous experience using computers or tablet PCs affected their ability to use the eSALT (v1.1) program. Age was also perceived by one participant as a limiting factor.

##### Category 2—support to use eSALT

Five factors were identified by the participants as having provided support for the use of the tablet PC and eSALT program: The initial training session, the user manual, family member support, support from the speech pathologist and the home visit (see Supplementary Table 1). The support provided through the initial in-person training session and by family members were the most dominant factors noted by participants. Participants reported that ongoing support was an important factor that affected their ability to access and use the system and contributed to the acceptability of the telerehabilitation approach. For this reason, most participants reported that the home visit during the trial was important.

All participants reported that support from family members would be useful, although the role of family members varied greatly between participants. Three of the five participants reported that the support of family members was important in assisting with technical issues. Of the three participants who lived alone, two reported contacting a family member for assistance with a technical problem. Three participants reported referring to the user manual during the home trial and that the user manual was helpful and easy to follow.

With regard to seeking remote support from the clinician, participants' views were mixed. Three of the participants reported seeking support from the clinician by phone or email when experiencing technical issues. While the other two participants expressed a desire to seek support from the clinician, they felt it was too difficult to make contact (e.g., use the phone).

##### Category 3—telerehabilitation

The participants explored the telerehabilitation aspect of the study and how remote monitoring influenced the usability and acceptability of the eSALT delivered therapy. Two sub-categories emerged from this category: Remote update of therapy tasks and the remote monitoring of progress (see Supplementary Table 1).

All participants viewed the remote transfer of therapy tasks positively. Participants expressed approval regarding the process of receiving new therapy tasks over the Internet and viewed the reduced travel time as a significant benefit of this telerehabilitation approach.

The degree to which participants valued the remote monitoring of their progress varied. Three participants enjoyed sending their data to the clinician and knowing their progress was being monitored. These same participants also expressed a desire for further feedback regarding their progress during the home trial. The other two participants' feelings toward the remote monitoring and feedback on progress were neutral.

##### Category 4—self-management

Within the category of self-management, participants' responses were coded into four sub-categories, which included intensity of practice, independence, motivation, and barriers to self-managing therapy. These are summarized in Supplementary Table 1.

All participants reported using eSALT daily and participants noted that greater intensity of practice was achievable as they had control over their practice schedule. Participants identified that the remote delivery of therapy promoted independence. However, several participants felt that at times independent practice was impeded by technical difficulties which they needed assistance to overcome.

**Figure 4:**
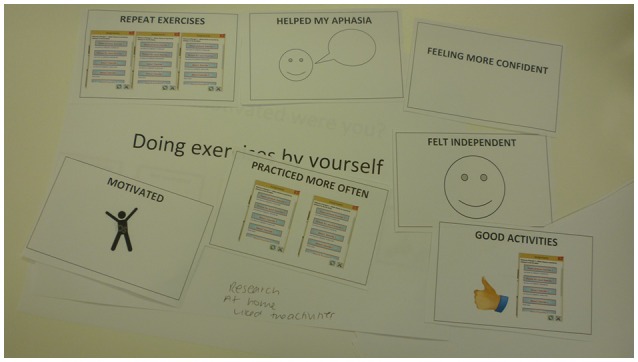
**P2's response to interview questions about accessing therapy independently**.

All participants reported being motivated to use eSALT, with the majority stating that this was due to the ease of accessing the therapy at home through eSALT (see Figure 4 for P2's results using communication support strategies). Another motivating factor identified by participants was the desire to improve their ability to use the tablet PC. Participants also suggested that their commitment to practice for half an hour a day was an intrinsic motivator. However, barriers to self-managed therapy practice were also identified and included reduced general wellbeing and technical problems.

##### Category 5—satisfaction

The final theme to emerge from the analysis was satisfaction, in which four sub-categories were identified: Satisfaction with the mode of service delivery, perceived beneficial outcomes, advantages of the telerehabilitation approach compared with in-person service delivery, and dissatisfaction with the therapy activities (see Supplementary Table 1).

All participants expressed satisfaction with the telerehabilitation approach and the eSALT (v1.1) program and all stated that they would like the opportunity to continue using it for therapy. However, levels of satisfaction with the personalized electronic therapy tasks varied greatly between participants. While some of the participants found the tasks easy to understand, others felt that the purpose of tasks was not clear and that the labels given to the activities by the clinician were difficult to understand. Participants also had mixed views on the grading of tasks. Some participants reported that the tasks were too difficult, while others considered the tasks too simple and desired something more challenging.

The participants described many perceived benefits of doing therapy tasks through eSALT. Participants perceived that the therapy activities improved their aphasia and assisted them to develop strategies to improve their communication. Increased confidence with communication and improved ability to use a tablet PC were also reported by participants.

In addition to the perceived beneficial outcomes, participants identified a range of advantages of remotely-monitored therapy compared to traditional in-person therapy. The most significant advantage identified by participants was that the telerehabilitation approach allowed for more intensive practice of therapy activities. Participants expressed a clear preference for the high levels of repetition provided by the eSALT platform. The ease of accessing therapy at home was highly valued by all participants.

### Clinician participants

#### Observational checklist

**Figure 5:**
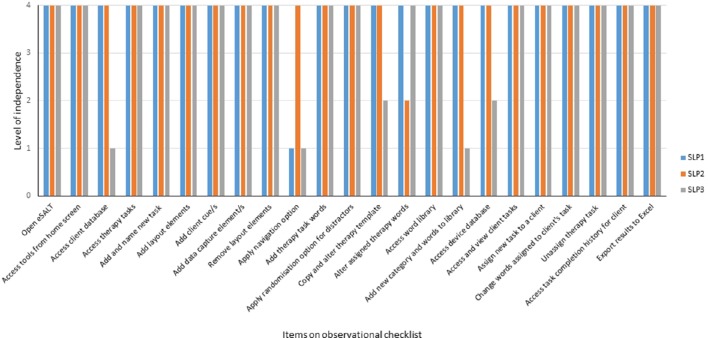
**Ratings of clinicians level of independence when using eSALT to design tasks and remotely monitor client progress**.

The results of the observational skills assessment are displayed in Figure 5. Overall, the clinicians were independent in their use of eSALT (v1.1) across 17 of the 23 skills. Where clinicians did not demonstrate total independence, they were either able to demonstrate the skill with assistance or were aware of the feature but had chosen not to use it in their development of tasks.

#### Clinician satisfaction survey

Each clinician participant completed the 54-item survey online. Results for the 30 5-point scale items are shown in Table 3. Overall the clinicians found the manual useful or did not use the manual during the trial. In terms of developing therapy tasks the clinicians were satisfied with the variety of task templates available in eSALT (v1.1) and generally found the steps involved logical. The clinicians stated that designing a task from scratch took 25–30 min to over 30 min. The clinicians' ratings of the cues available in eSALT (v1.1) (Q.9) and the data capture elements (Q.11) were mixed and reflected the different usage of eSALT (v1.1) by the clinicians, such as not using the cues in their tasks or not being familiar with reading data in Excel. Mixed ratings were given on the question of importing salient images into the word library (Q.18) due to this feature being disabled during the trial and so clinicians did not use this feature. SLP3 disagreed that there were a variety of randomization methods available in eSALT (v1.1) (Q.19), commenting that the target word was not able to be randomized when assigning the task to the client. With regard to using eSALT (v1.1) to remotely monitor the participants' progress, the clinicians generally agreed with the statements about ease of assigning tasks to participants and viewing data. Two of the SLPs were neutral in their response to how successfully they could view their participant's progress on a specific task (Q.22) and whether they enjoyed monitoring their participant's progress in this way (Q.26). Two of the clinicians reported that they spent 20–30 min monitoring and updating tasks for each participant, while the other clinician reported spending 60 min on these tasks. Overall, the clinicians were satisfied with eSALT (v1.1), felt it would be useful with their current aphasia caseload as well as other communicatively disordered populations and would be likely to recommend it to other SLPs.

**Table 3:** **Clinicians' ratings on satisfaction survey**.

**Statement in satisfaction survey**	**SLP1**	**SLP2**	**SLP3**
**MANUAL**			
1. The manual provided was useful	3	4	4
2.The manual assisted me in using eSALT	3	3	4
3. I was satisfied with the manual provided	3	4	4
**DEVELOPING THERAPY TASKS**			
4. The steps to develop a therapy task were logical	4	3	4
5. I found developing a therapy task confusing or difficult	4	3	4
6. eSALT was flexible in terms of designing a variety of tasks	4	3	4
7. eSALT provided a good variety of task templates.	4	4	4
8. eSALT was flexible in terms of customizing tasks for individual clients (e.g., levels of difficulty)	3	3	4
9. eSALT contained cues which covered the majority of your needs	4	3	2
10. eSALT contained a good variety of data capture elements	3	4	3
11. The data capture elements provided me with useful data	2	3	2
12. It was useful having a mock-up of the task layout	5	4	5
13. Overall, I found using eSALT to develop tasks time consuming	4	4	4
**STIMULI**			
14. eSALT contained culturally appropriate stimuli in the word library	5	5	3
15. eSALT contained an adequate number of stimuli in the word library	4	4	5
16. eSALT contained high quality stimuli (e.g., appropriate for adults, clear and unambiguous images)	5	5	5
17. eSALT allowed client images to be imported into the word library to increase saliency.	2	4	3
18. A variety of randomization methods to present stimuli were available in eSALT	5	4	2
**TELEREHAB MODULE**			
19. It was straightforward/easy to assign therapy tasks to the client	4	4	4
20. eSALT allowed me to view how often a client did each task	4	4	4
21. eSALT allowed me to view how successfully my clients completed their therapy tasks.	3	3	4
22. eSALT allowed me to export raw data to Excel	4	4	4
23. I used the data captured by eSALT to make judgments about upgrading/simplifying therapy tasks.	3	4	4
24. I liked monitoring my client's treatment and progress this way.	3	3	4
**GENERAL eSALT**			
25. It was easy to navigate between functions in eSALT	5	4	4
26. The time spent initially developing the therapy tasks was worth it as it resulted in ease of use in the monitoring and updating of tasks.	4	3	4
27. This software would be useful in the delivery of therapy to my current clients with aphasia in my workplace	5	4	4
28. eSALT would be a useful software program for use with other communication disorders	5	4	4
29. I would be likely to recommend eSALT to other SLPs	5	4	4
30. Overall, I was satisfied with this software tool	4	4	4

#### Semi-structured interviews

Content analysis of the SLP's interview data revealed six main categories: (1) using eSALT; (2) preparation of therapy tasks (3) remote monitoring; (4) overall satisfaction; (5) applicability and (6) benefits of eSALT. These categories directly addressed the usability (categories one, two, three, and four) and acceptability (categories five and six) of the system. See Supplementary Table 2 for summary of results.

##### Category 1—using eSALT

Within this category of using eSALT, the SLPs' responses were coded into four sub-categories: Interface was easy to use, learning to use, efficiency, and familiarity with program improved usability.

The SLPs felt that the interface was easy to use and that learning to use eSALT (v1.1) was also easy and did not take too much time. The SLPs considered the in-person training in the use of eSALT as important and that the user manual was also useful. However, all of the SLPs reported that the eSALT (v1.1) program took too long to perform certain actions, such as saving and uploading tasks and that this inefficiency was frustrating. Nevertheless, upon reflection one of the SLPs felt that the time taken to use eSALT (v1.1) was probably comparable to traditional therapy preparation. The SLPs also reported that as their familiarity with the program increased so did their effective and efficient use of the program (see Supplementary Table 2).

##### Category 2—preparation of therapy tasks

One of the unique features of eSALT is the capacity to develop and fully customize therapy tasks, therefore a major category related to the preparation of therapy tasks and included six sub-categories: Development of tasks from scratch, copying and personalizing tasks, variety of tasks, usability with variety of aphasia types currently restricted, stimuli and cues and the time taken to develop tasks (See Supplementary Table 2).

All of the SLPs reported initial difficulty developing therapy tasks from scratch, with one SLP stating “I think to develop from scratch there are several steps that you need to step through and get them all right before you can upload to the tablet.” In contrast, all of the SLPs reported that copying a task template and personalizing it was easy and straightforward. On the topic of the task templates, all of the SLPs felt there was a good variety of templates, but that a larger bank of task templates would improve the program. This idea of a larger bank of therapy task templates was also related to being able to use eSALT with a wider variety of aphasia types, in that there were few high level language task templates available. Furthermore, limitations in the (v1.1) program, such as restrictions on the amount of text able to be displayed and the use of punctuation in tasks, impeded the development of higher level tasks by the SLPs.

The SLPs also commented on the time it took to develop tasks, expressing some frustration with the amount of time spent developing tasks. However, all of the SLPs felt that there was great variety and high quality stimuli available in eSALT (v1.1) and that the stimuli were culturally appropriate. They also commented on the categorization of the stimuli and how that enhanced the usability of the program. Likewise, the SLPs reported that the cues available were useful.

##### Category 3—remote monitoring

This category contained three sub-categories: Assigning tasks and stimuli, reading and using data, and SLP/client contact (See Supplementary Table 2). All of the SLPs reported that assigning tasks to clients was easy and that updating stimuli within the tasks was also easily achieved. It was felt that this process of assigning tasks or stimuli was efficient. However, one of the SLPs cautioned that clinicians need to be aware of the potential to assign too many tasks to an individual.

The SLPs all reported that reading the data in Excel was difficult and time consuming and that ultimately they didn't use that data during the trial. However, the SLPs liked the display of data in the calendar and used this data to make a number of decisions about the number of tasks to assign to an individual, the appropriateness of the tasks and the level of difficulty and when to remove a task.

The final sub-category was SLP/client contact. Face-to-face contact with the participant with aphasia before the use of eSALT was considered important, as was continued contact with the participant with aphasia during the eSALT trial. One of the SLPs developed her own method within eSALT (v1.1) to let the participant with aphasia know how to contact her.

##### Category 4—overall satisfaction

Within the category of overall satisfaction, codes were grouped into three sub-categories: Perceptions, experience using eSALT (v1.1) and future use (See Supplementary Table 2). Despite the SLPs' expressing frustration at the time inefficiencies within eSALT (v1.1), the SLPs' perceptions of eSALT were generally positive with comments such as “I think it's a great program at the end of the day”. Two of the SLPs felt that eSALT was cutting edge and impressive. The SLPs reflected on their experience using eSALT (v1.1) positively, using terms such as “interesting” and “fun.” With respect to potential future use of eSALT, all of the SLPS stated that once fully developed eSALT would be a great therapy tool. Two of the SLPs stated that they would use it again and one felt that her workplace would be interested in such a program once available.

##### Category 5—applicability

The category relating to the applicability of eSALT contained three sub-categories: Clinical settings, client considerations and service delivery (See Supplementary Table 2). All of the SLPs reported that eSALT could be applied across a range of clinical settings, including inpatient, outpatient, and community rehabilitation settings. It was also considered ideal for continuing therapy with rural clients.

The SLPs did, however, feel that certain client factors would need to be considered when using eSALT. These factors included client's motivation, client familiarity with technology, any co-morbidities they may have and the availability of support at home to use eSALT. Nevertheless, one SLP felt that eSALT could be used with a wide variety of clients, not just those with language impairment.

Despite the SLPs in this study only using eSALT in an asynchronous telerehabilitation service delivery model, they discussed how eSALT could be used in other clinical service delivery models. Two of the SLPs thought that eSALT could be used in traditional in-person therapy sessions, as an adjunct to paper-based therapy, or with support from therapy assistants across the day. In the context of home-based use the SLPs felt that eSALT could be a tool to encourage family involvement in therapy or could be used to provide homework tasks between clinic visits. One of the SLPs talked about the capacity to remotely monitor client's progress and then adapt tasks in a more timely manner.

##### Category 6—benefits of eSALT

The final category that emerged from the analysis examined the benefits of eSALT, with codes relating to benefits both for people with aphasia and clinicians (See Supplementary Table 2). For people with aphasia, the SLPs thought that eSALT contained a number of benefits over traditional worksheets, such as dynamic cueing, more salient and personalized therapy tasks, and greater variety of tasks which would maintain the client's interest. Additionally, the SLPs felt that eSALT was modern, culturally appropriate and less isolating for people with aphasia.

With regard to their own use of eSALT, the SLPs reported a number of benefits. These benefits included the ability to change tasks quickly and more frequently, as well as easily adjusting the level of task difficulty. Some of the SLPs felt that it was beneficial to provide clients with electronic tasks as the results are calculated for the clinician. The range and type of stimuli available in eSALT was seen as giving the clinician the capacity to quickly and easily provide tasks for clients.

##### Suggested improvements to eSALT

During the interviews the SLPs made some suggestions for improvements to eSALT (v1.1). These direct suggestions were extracted from the interviews and are summarized in Table 4.

**Table 4:** **Improvements to eSALT suggested by clinician participants**.

**Suggested improvement**	**Supportive quote**
Customize the menu order for the patient	Even if I could have just ordered it I think in a different way or perhaps had the ones that they were supposed to do that day come to the top of the screen
Go through task before giving to client	I would actually like to go through the task before I actually gave it to them.
Message system built into eSALT	Somewhere communicating with them through the program would be good as well, so a little messenger system like “How are you having problems? Or How did you find today?”
Expand library	so maybe some more abstract words
Expand task templates	I think there could be more, especially for the higher level patients
Filing of task templates	I think there needs to be some way of filing that bank of tasks instead of just one big line alphabetically listed.
Randomization of target	I found that everything was really presented in alphabetical order, and if there was any way within the task assignment section to then randomize items, so that you could choose the first one to 20, and it wasn't in alphabetical order.
Display record of assigned tasks and items	if there was some kind of way to keep track of the assigned verses the unassigned items, when you're re-uploading new items within the same task.
Summary of data would be useful	but it would have been nice to have a summary as well, just something that was quite—they got this out of this right and this is how long it took them to complete it overall
Video of how to rather than manual	Probably more than a manual would be helpful. Maybe a DVD showing how to do a task so that you could stick it into another computer and watch someone doing a task while you did it with them would be good.
Forum for SLPs to share task design ideas	Would be good if it could be in some sort of forum or if you had all the clinicians in your clinic all adding to the same bank of information so that after a while you'd have everyone's ideas on there and somewhere to share them and add pictures and stuff.
Highlight side of screen for neglect	You could have a left and right side neglect and so traditionally you'd get a highlighter out and highlight down the side of the page. So maybe if it's possible to highlight parts of the page.
Variable transition time on tablet	When I was doing it myself at home, I thought is so slow I wish it would go faster. But actually, for the client themselves, it was too quick.
Expand audio cues	I found that the audio cue said the whole word and it would have been nice to just have the initial sound.

## Discussion

This study sought to explore the usability and acceptability of a prototype asynchronous telerehabilitation platform, eSALT (v1.1), from the perspective of both people with aphasia and clinicians, with a view to further refining the platform. Results from a range of outcome measures indicated that while the prototype was usable, there were a number of key improvements required in order to enhance the usability and acceptability of the platform for both end-user groups.

### Usability and acceptability of eSALT from perspective of people with aphasia

As found by both Galliers et al. (2012) and Simic et al. (2016), the end-user design approach was successful in identifying features of the program that should be modified to enhance the usability and acceptability of the software for the users with aphasia. Furthermore, the collection of usability and acceptability data from both observation in a real world setting and interview provided additional confidence that the most important areas were identified for refinement. Simic et al. (2016) also employed this combination of data sources in their usability study of a synchronous telepractice system for anomia therapy. As in these previous studies, the use of communication supports during the interviews enabled those participants with more severe expressive difficulties to participate in providing their perspective and so provided a rich data set (Galliers et al., 2012; Simic et al., 2016).

In terms of usability, both sources of data (the independent observations of the participants accessing therapy tasks and qualitative data from the interviews) revealed that the majority of the participants were able to successfully and independently use the tablet and the program. However, some skills such as using the scroll bar and using different types of input types (e.g., keyboard) were found to be problematic for some users. For some participants these difficulties arose due to the variable responsiveness of the touchscreen on the devices, while other participants were observed to press too hard on the touchscreen. It may be that these access issues were partly due to the participants' inexperience with the mobile device or insufficient training; however it is also important to consider that co-morbidities such as reduced cognitive ability or impaired dexterity may also impact on a user's ability to access a program. Tasks which required fewer steps such as “open therapy task from list” and “use synchronize icon on eSALT menu to send results” appeared to be easier, which may be due to the fact that they place fewer demands on working memory, cognitive and language skills.

Nevertheless, it is essential that appropriate hardware be used and that usability testing extend from the software to also include the hardware. This prototype of eSALT (v1.1) was developed for use on a Window mobile device and the devices used were mid-range products rather than premium products. Thus, the sensitivity or responsiveness of the touchscreen may not have been ideal. Additionally, in a Windows operating system the standard method of accessing off-screen items on the main menu is via a scroll bar whereas swiping is the standard method used on an Apple iPad®.

During the interviews the participants with aphasia identified a number of factors which they felt influenced their use of eSALT (v1.1) and the tablet. While some participants felt that personal factors such as age or experience with technology may have been influential, all participants agreed that training and support were crucial factors in their use of the program. Research supports this finding, with Kurland et al. (2014) reporting that adequate training and motivation were more important predictors of successful technology use than age, aphasia severity or previous computer experience in their trial of a tablet-based therapy program. With regard to training and support, the participants in this study especially valued the initial in-person training and the subsequent home visit in which additional personalized training or troubleshooting took place. Other researchers have also identified training and ongoing support as crucial factors in promoting independence and competency in operating computer-based aphasia therapy programs (Egan et al., 2004; Kiran et al., 2014). Personalized support was also needed by the participants when technical problems arose. It was interesting that some participants did not want to contact the SLP or researchers when faced with technical problems, but rather contacted family members to ask for assistance. As the use of technology in aphasia rehabilitation grows, a key consideration should be how best to provide tailored technical support to people with aphasia, especially those without personal support networks.

Interestingly, the participants in this study had mixed views as to whether the remote monitoring of their progress by a SLP was a motivating factor. This is in contrast to other studies which have reported the monitoring role of speech pathologists to be a crucial and highly motivating factor in self-managed computer therapy (Wade et al., 2003; Palmer et al., 2013). Those participants who did want to know how well they were progressing through the therapy tasks suggested that this could be achieved either through direct contact with the SLP (e.g., telephone call) or through the eSALT program itself if results could be displayed to the user.

In terms of acceptability of the program and the asynchronous telerehabilitation model of service delivery, all of the participants expressed high levels of satisfaction with the use of eSALT (v1.1) and the asynchronous telerehabilitation model. High levels of participant satisfaction are commonly found in telerehabilitation studies and have also been cited in other usability studies involving telerehabilitation (Simic et al., 2016). As in the Wade et al. (2003) study participants in this study reported high levels of motivation to practice intensively and reported that this was achievable because they could access the therapy at home and had autonomy over their practice schedule. The participants highly valued the personalization of therapy tasks that was achievable through eSALT (v1.1) and appreciated the wide variety of tasks available to them through the program. However, some participants felt that the tasks were not well matched to their needs. This is an interesting finding given that eSALT (v1.1) had the capacity for therapy tasks to be individually tailored to a client's specific needs. This mismatch may have been due to a limitation in the research design, as the clinicians only had one assessment session with participants before designing the tasks, or it may have been due to the clinicians' not receiving enough training in the use of eSALT (v1.1). A trial which more closely imitated a clinical setting in which the clinician and participant met in-person for several sessions may have allowed clinicians to determine whether the tasks designed for participants were appropriate and make more changes in response to participant feedback. This need to appropriately align therapy tasks to the client's skill level was also identified in the usability study by Simic et al. (2016).

In summary, the results from the participants with aphasia confirmed the acceptability of the asynchronous telerehabilitation service delivery achieved through eSALT (v1.1), but identified a number of improvements which would enhance usability and acceptability of the program. The major improvements identified included changes to the mobile device used, removing the need for a scroll bar, personalization of the task menu and provision of additional immediate feedback to the user on therapy progress.

### Usability and acceptability of eSALT from perspective of clinicians

The recruitment of practicing SLPs and the embedding of this usability and acceptability study in a clinical scenario yielded rich data for the refinement of eSALT (v1.1) program. To the authors' knowledge this is the first study to involve SLPs in an end-user design approach for an asynchronous telerehabilitation platform and specific to the clinicians' use of the program, although clinicians have been involved in usability studies of synchronous telerehabilitation systems (Simic et al., 2016). In both the current study and the Simic et al. (2016) study clinician participants had no prior experience of the telerehabilitation system being evaluated. A potential strength of this sampling method is that the data gathered is ecologically rich and possibly more generalizable to clinical practice.

In terms of the usability of eSALT (v1.1) the clinicians identified a number of strengths of the program, including the layout of the interface, the quality, appropriateness and variety of stimuli available, the variety of task templates and the ease with which they could personalize these templates for their participants with aphasia. However, the clinicians found designing therapy tasks from scratch difficult and time consuming, and even more so for higher level language tasks. They expressed frustration at the time the program took to save, which they felt impeded the acceptability of the program overall. One factor in the poor efficiency of the eSALT (v1.1) program was that the hardware used had low processing power and memory. It is important that the hardware chosen is fit for purpose so that usability data is unambiguous in terms of its relationship to the software or hardware. Future studies should ensure that optimal hardware is utilized. Nevertheless, refinement of the eSALT (v1.1) program's efficiency was a key finding.

Another key finding for the usability and acceptability of eSALT (v1.1) related to reading and using the data from the participants with aphasia. As evident from the satisfaction survey and the interviews, all of the SLPs in this study found reading data in Excel spreadsheets difficult and did not use this data in their decision making. However, they liked the simple visual display of data in the calendar and used this data to make decisions about the appropriateness of tasks and when to update tasks. These decisions were based on the participant's completion of tasks rather than success or accuracy in completing the tasks. Improvements to eSALT in terms of summation of data and graphical display of accuracy data should enable clinicians to more easily use the data capture features that this telerehabilitation platform offers, thus enhancing its usability and applicability clinically. The capacity for clinicians to use data to make clinical decisions about therapy progress is crucial to the successful uptake of asynchronous telerehabilitation.

Contact between the SLP and the participant with aphasia was also considered a key factor in the success of an asynchronous telerehabilitation service delivery model. While one clinician used the eSALT (v1.1) program to display contact information to her participants, it was felt that a specific messaging system within the eSALT program would enhance its usability and acceptability.

In terms of the acceptability of the asynchronous telerehabilitation service delivery model enabled through eSALT(v1.1), the clinicians believed that the eSALT platform was applicable in a number of clinical settings and could be used in service delivery other than asynchronous models, such as during in-person therapy sessions. These findings indicate high levels of acceptability for eSALT and highlight the flexibility of this asynchronous telerehabilitation platform. Other studies have also highlighted the capacity to use asynchronous telerehabilitation systems in clinical sessions (e.g., Des Roches et al., 2015). All of the clinicians in the current study stated that they would use eSALT again and also recommend it to others once further refinement of the program was complete (See Table 3). The clinicians anticipated many benefits for people with aphasia using eSALT, including improved access to intensive therapy practice, therapy that is more salient through the personalization possible in eSALT and greater variety in therapy tasks. For themselves, the clinicians felt that eSALT enabled them to provide more salient therapy and to easily and more frequently change the tasks given to a client. They also believed that with further development the use of data from eSALT would improve their capacity to provide personalized therapy to their clients.

### Refinement of eSALT to enhance usability and acceptability

The usability and acceptability data from the interviews with both end-user groups was combined with the suggested improvements (see Table 4) to identify and prioritize the refinement of eSALT through a process of feature mapping. Table 5 summarizes the refinements identified, the current status of the eSALT platform and proposed future development of eSALT.

**Table 5:** **Feature mapping from usability results to current functionality of eSALT and future development**.

**eSALT area**	**Improvement from usability study**	**Current functionality (v4.2)**	**Proposed future development**
Clinician user experience	Categorize therapy task templates Record of words assigned to each client. Randomization of target presentation Reduce time to save and load. Extend amount of text allowed and punctuation. Preview device experience during task development. User uncertain of steps needed to develop tasks Graphical summary of client results	Homepage dashboard developed to view latest results for current client. Task development - Target setting for all task inputs to compare with results returned and generate score for each exercise. - Text length aligned to device viewing limit and most English punctuation allowed. Task Assignment screen re-designed - Graphical interface for easy visualization of usage of words in each task and drag and drop editing. - Task words/pictures can be distributed randomly into multiple assignments. - Preview option provided to step through therapy task. Client monitor screen developed specifically for review of client progress - Numerous performance graphs for clients per task and over time. - Export of data for further analysis. Changed data storage and access method to improve efficiency of program	Categorization of tasks (e.g., Reading, Writing, Listening, or Speaking). Options to view only their own tasks, imported shared tasks, or all tasks. Capacity to attach multiple free-form tags to tasks. Typical steps in task development and beneficial features will be exposed via a wizard. User interface will continue to be improved to support ease of use and increase responsiveness. Accept punctuation beyond English and move toward a language-independent program. The preview feature will be adapted to match the variations in device layouts to ensure that the clinician is confident about what the client will see on their device.
Client user experience	Scrolling on touch screen difficult Unsure about single or double click on icons. Did not know what to do with new tasks. Visual cues for oral placement. Transition time between items was too quick. Feedback on progress desired Option to have whole word or initial sound cues.	Developed client app for iPad - Simpler, more familiar interface. - Improved user interaction (e.g., touch response, swipe action) Considerable improvements in setup and synchronization process (e.g., automatic transfer of results) Notification to user when new tasks are available for download. Navigation option disabled and animated feedback when transitioning. Graphical feedback of score to user	Include support for Android devices. Clinician-controlled tailoring of device behavior to client's abilities and needs to be developed, including the ability to specify what content is presented in the cues such as initial sound or full word. Feedback to client will be extended to show their progress toward their goals.
Clinician and Client Interaction	Customize the menu order for the client. Client did not understand the names/jargon of the task. Capacity for communication between client and SLP desirable	Device menu can be fully modified to specify menu order, task labels and combine tasks under one menu item. Messages can be attached to a task for display when task is next done. Instruction or feedback forms can be created to communicate with patient.	Clinician will be able to select some device behavior and media content to match client's abilities and needs. The tailoring that exists in the application such as device menu management will be made more transparent to the user.

The major usability issues identified by the SLPs were addressed through a number of key developments. Firstly, efforts to improve the efficiency and responsiveness of the eSALT (v1.1) platform were actioned through migration to a more efficient database, the upgrading of hardware and streamlining data access. In order to assist with task development and improve the ease with which clinicians could give a range of clients with aphasia appropriate tasks, the list of task templates was substantially expanded to over 50 tasks, including high level language tasks. Restrictions on text length and punctuation were relaxed and the word/picture library was expanded to over 1300 high quality color photographs across a wider range of categories (e.g., verbs, money). Custom images can now be remotely uploaded to the cloud by the administrators for download to the desktop and mobile device as required.

**Figure 6:**
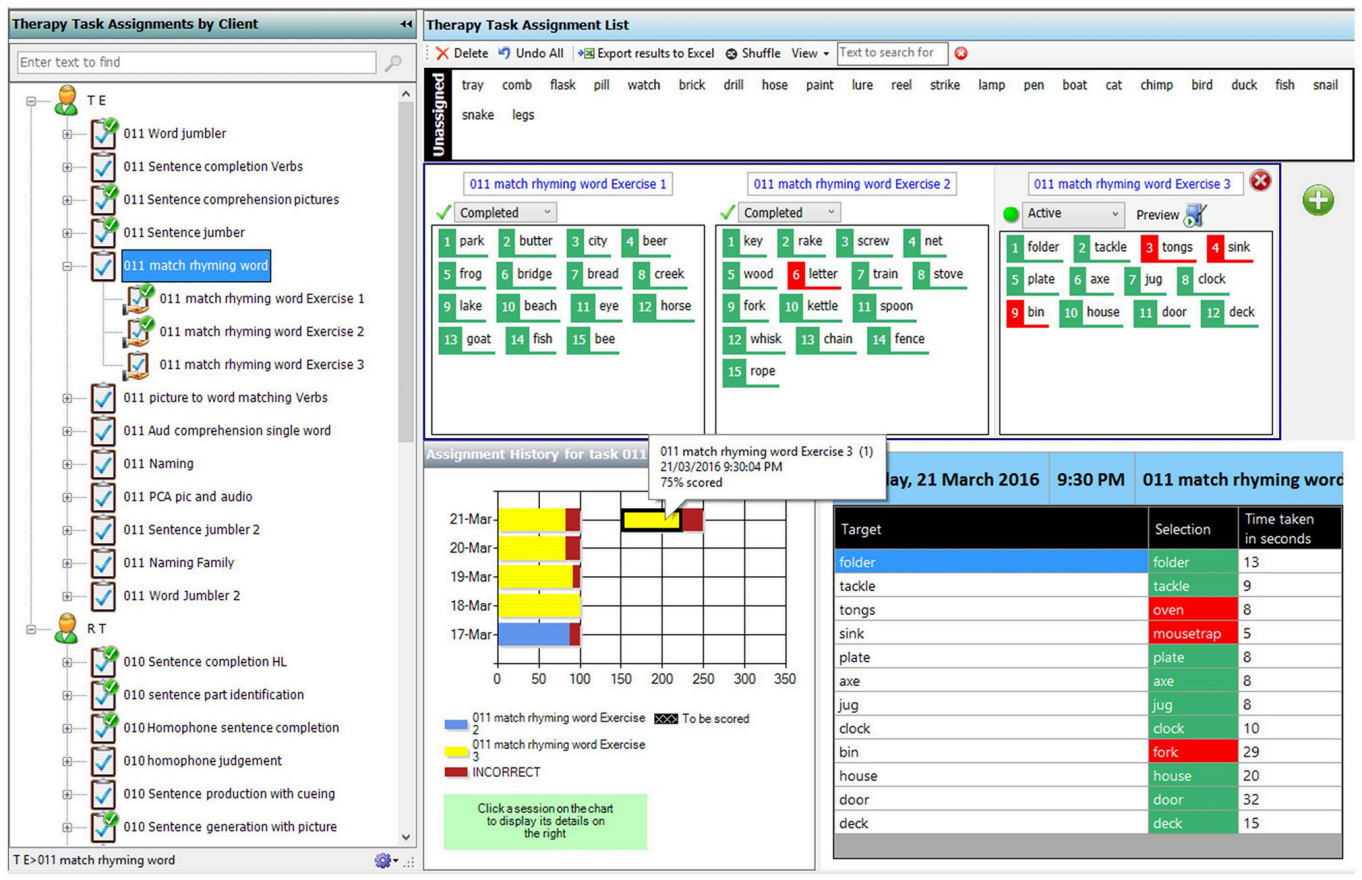
**Redesigned therapy task assignment screen in salt 2015**.

In order to facilitate efficient feedback on client progress to the client and the clinician, the capacity to set the target response within a task was expanded to all response inputs (except free text input). This key development enabled results, such as percentages correct, to be displayed to the client at the end of each task and also assisted with automatic scoring and summation of client progress for the clinician. This automatic scoring functionality may further improve the accessibility and usability of eSALT for both clients and clinicians, as it should significantly reduce the time clinicians need to manually score tasks and provide feedback, as well as, provide the option for immediate feedback to the client. However, it is up to the clinician to input these correct responses thus maintaining the flexible functionality of eSALT and aligning with the the recommendations arising from the Swales et al. (2016) study which found that clinicians wanted to tailor feedback options for individuals. Functionality around the assignment of words/pictures to tasks and tasks to clients was also improved through the re-design of this interface to a more graphical layout which enabled randomization of words/pictures into exercises and also allowed for a step-through preview of the tasks in eSALT (v4.1 onwards) (See Figure 6).

**Figure 7:**
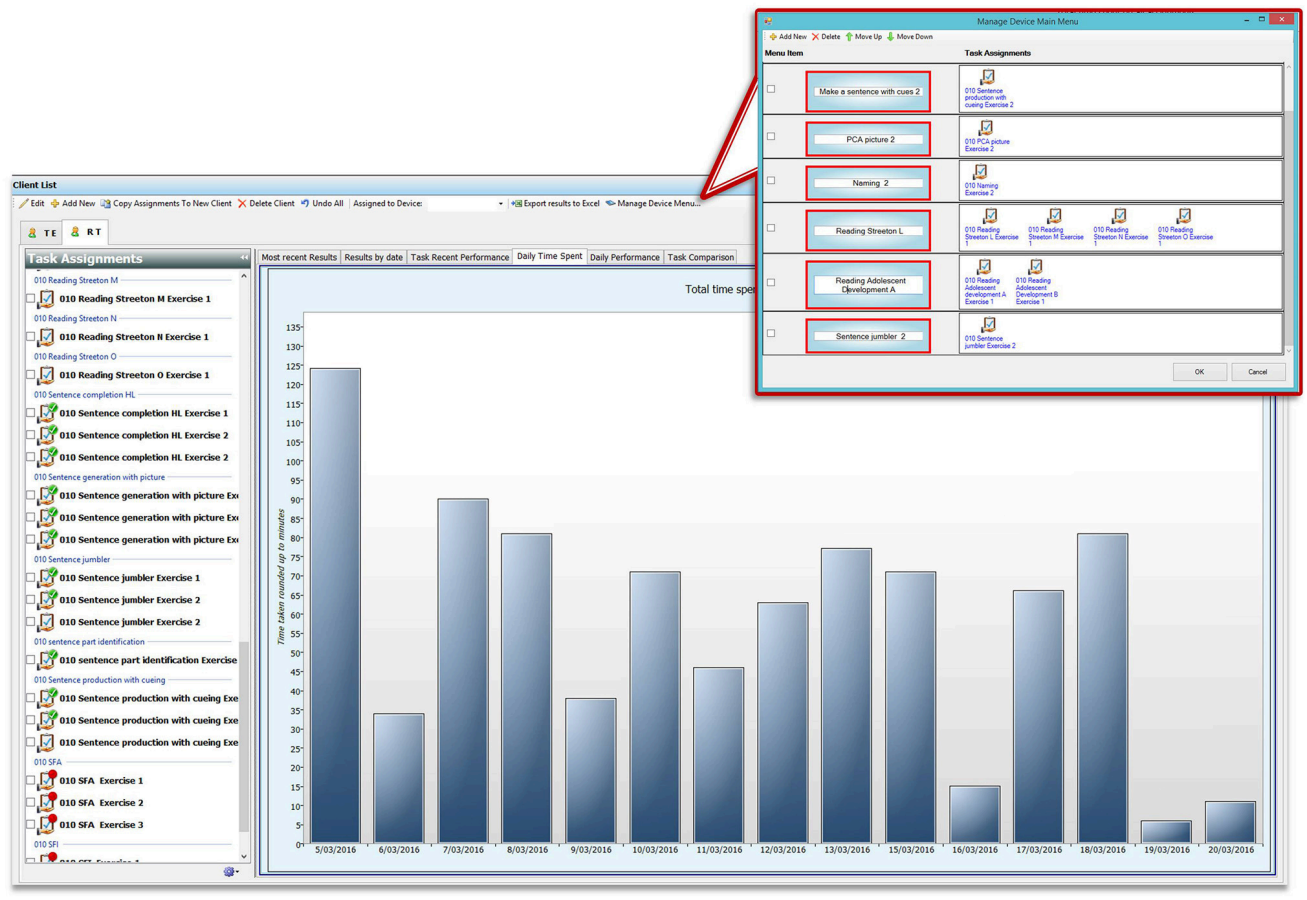
**Additional client monitoring interface in eSALT 2015**.

Feedback from the SLPs about the difficulties reading and using client data led to the development of an additional interface specifically for monitoring client progress (eSALT v4.1 onwards). This interface provides the clinician with numerous graphical displays of client data, both per task and over time. The capacity to export data to Excel remains active. This client monitor interface also enables further customization of the display of tasks on the client device, such as changing labels on the task menu and the order of tasks in the menu (see Figure 7). However, perhaps the most significant development in this interface was the capacity to combine different tasks under one menu label. This feature enables the clinician to have an initial page which may explain the task, joined up to the task itself. This also enables reading comprehension tasks to be designed with multiple pages of diverse text and questions within a single task. Another feature of this interface is the ability to attach messages either to a specific task or as an overall message to the client. Overall messages may be designed with questions for the client to complete, thus supporting communication between the clinician and client.

**Figure 8:**
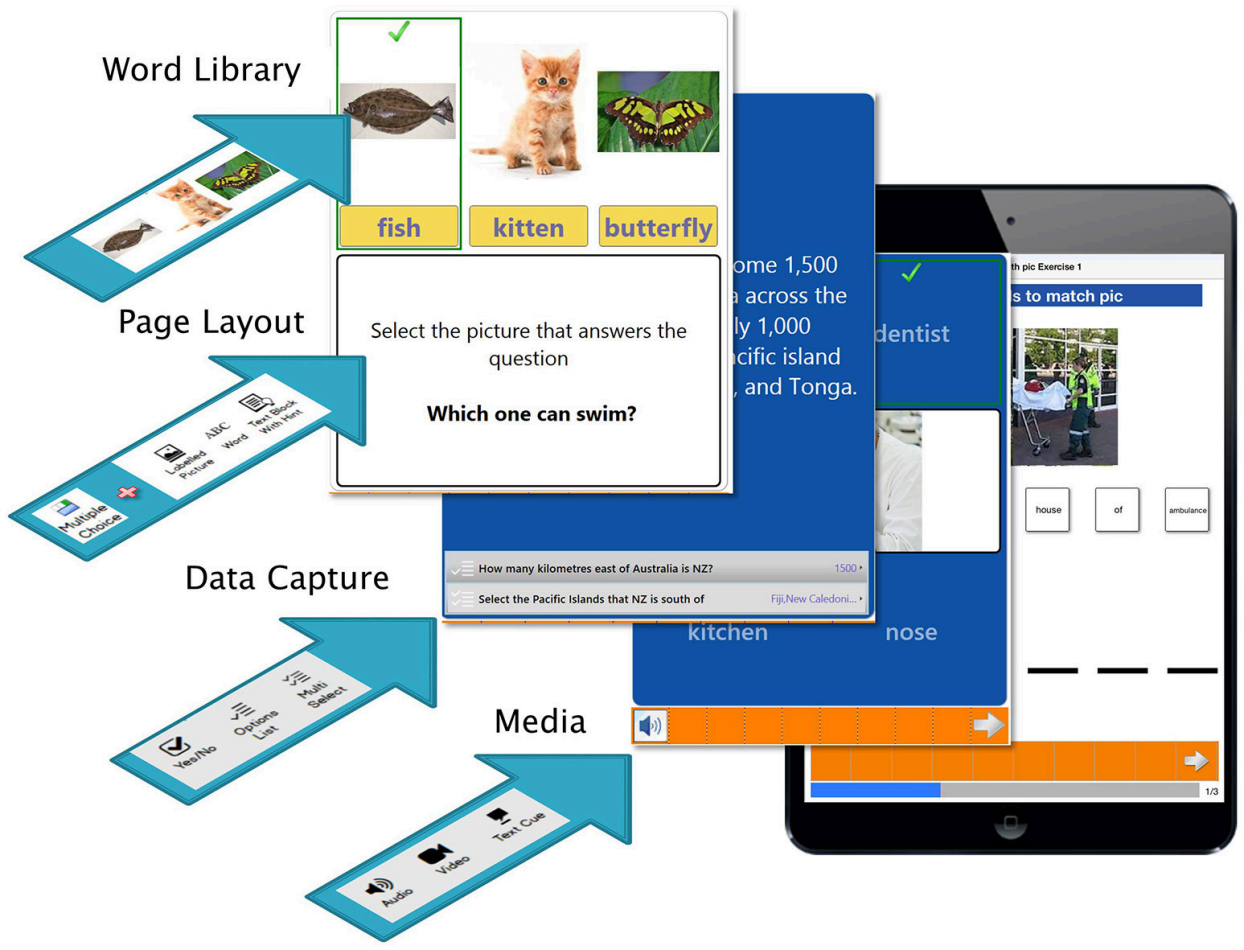
**Samples of preview of iPad layout from new task design screen in eSALT 2016**.

In response to the usability data from the participants with aphasia, a client application was developed for the Apple iPad® (see Figure 8). This development expanded the use of eSALT (v4.1 onwards) across a two operating systems (Windows and iOS) and in doing so goes some way to fulfill a recommendation that arose from the Swales et al. (2016) study that software be available across multiple platforms. The iPad® application has a simpler interface and standardized user inputs (e.g., 2 step menus for input of single or multiple selection response). User interactions such as the touch response and swipe action also improved with the move to the iPad®. In terms of the user experience, transfer of task results was automated and users are now notified when new tasks are available on the server for download to the device. Users can no longer inadvertently skip through task items and users can now receive graphical feedback of their score as a task is completed.

#### Future development of eSALT

Proposed development of eSALT outlined in Table 5 will continue to focus on improving areas identified from end-user input and recommendations from previous studies. To assist clinicians in determining the ongoing progress of clients, individual usage goals such as daily time spent on tasks will be able to be set at client and/or task level. Progress toward each of the goals will be shown as a snapshot of overall performance and reported in a calendar view with a daily summary status. The homepage dashboard will become customizable so that a clinician can quickly see their preferred measures. A history by word or phrase will be maintained so that a profile of client capability can be built and used by the clinician to set the difficulty of a task. Feedback to the client on the device will be extended to show their progress toward their goals. Further development will include catering for a range of client abilities and needs through clinician tailoring of the device behavior and layout. To further improve the clinician experience, typical steps and beneficial features will be made more transparent and a high level of task categorization will be allowed. By the introduction of a web-based portal for the eSALT community, clinicians will be better able to enjoy the full potential of the system's flexibility through self-management and sharing of custom media and tasks. Finally, the authors plan to further expand the platform such that it is applicable across a variety of communication disorders and a variety of settings. One important expansion will be the capacity to transfer audio and video recordings to the clinician.

### Limitations and future directions

Although this formative usability study used multiple methods to collect data during a feasibly study, a number of limitations were evident. Firstly, the usability data gathered from both end-user groups could have more comprehensively covered the use of the software by use of an expanded observational checklist that required all potential uses to be observed (e.g., an item specific to the participant's use of the audio recording and playback function) or by the software itself tracking usability in the form of error counts. The study by Simic et al. (2016) collected error counts, however, this was achieved through observation rather than software tracking. Usage data detailing exactly how long each participant engaged in therapy using eSALT (v1.1) and how long clinicians spent designing tasks and monitoring progress was not available in this study. The capture of usage data will be vital in future studies to bestow additional credibility to the usability and acceptability of data collected. Furthermore, accurate usage data from both end-users would enable analysis of the potential cost-efficiency of asynchronous telerehabilitation in relation to the ratio of practice time by the participant with aphasia to clinician time in designing, monitoring and updating therapy tasks.

The hardware chosen on which to run the clinician's desktop application was not ideal, with insufficient processing power and memory for the complex processing requirements of such an open and flexible software program. This led to the program being inefficient in the performance of functions such as saving and loading words/pictures. It will be important that future usability studies ensure that hardware is fit for purpose.

The sample size of this usability study may be considered small, however, usability literature suggests that formative usability studies involving early prototypes and set in a real world context may record valid data from as few as five participants (Macefield, 2009). Nevertheless, given the heterogeneity and complexity of aphasia there may be a need for future usability studies to include a larger sample of people with aphasia as well as a larger sample of clinicians from a variety of rehabilitation settings.

Future usability studies should aim to further embed the research in real world settings so as to increase the ecological validity of the data and discover unanticipated issues earlier (Genov et al., 2009). For example, ecological validity of the data may be improved by including participants who are currently receiving other therapy, so as to diminish any potential Hawthorne effects in the data. Furthermore, there may be a need for different types of usability studies, such as comparative studies which might compare the usability of two or more interfaces or punctuated studies which promote an iterative development process (Macefield, 2009).

Since the completion of this study in late 2013, improved versions of eSALT have been used in a number of other research projects, including a trial of the effectiveness of the therapy provided through eSALT (UQ ECR Grant eSALT v4.2) and as part of a larger randomized controlled trial comparing a comprehensive intensive aphasia program to usual care (NHMRC Grant APP1057047 eSALT v4.1).

## Conclusion

This article introduced a novel asynchronous telerehabilitation platform for speech-language pathology, eSALT, and reported on how the results of a usability and acceptability study with two end-user groups (people with aphasia and clinicians) were used in the refinement and further development of the platform. Acceptance of eSALT for the asynchronous telerehabilitation of aphasia was confirmed by both the participants with aphasia and clinicians. While the platform was considered usable, further refinement was required in order to enhance the usability. eSALT will continue to be developed in accordance with the end-user design approach.

## Ethics statement

University of Queensland Behavioural and Social Sciences Ethical Review Committee. Participants were provided with an information sheet outlining the details of their involvement as well as verbal explanation of what their participation would involve. All participants were given the opportunity to ask questions of the researchers and were also given the opportunity to have a close relative or friend present. Then, written, voluntary consent was obtained from all participants prior to the commencement of the study. All participants were informed that they were free to withdraw from the study at any time without providing a reason and without it negatively affecting their relations with the University of Queensland. Separate information and consent forms were developed for clinician participants and the participants with aphasia. The information sheet for the participants with aphasia was formatted according to the aphasia-friendly guidelines outlined by Rose et al. (2011). If required communication support techniques as outlined by Kagan (1998) were employed to facilitate understanding and ensure informed consent was obtained.

## Author contributions

AH contributed to the design of the software described in the manuscript, the design and management of the research studies herein reported, interpretation of the data in relation to further development of the software and research findings, drafting, and revision of the manuscript. HB contributed to the design and development of the software described in the manuscript, interpretation of the data in relation to further development of the software, production of technical illustrations in the manuscript, and drafting and revision of the manuscript.

## Funding

The usability study outlined in the manuscript was supported by an Australian National Stroke Foundation Small Project Grant 2013 (014423) and an Australian National Stroke Foundation Honours Project Grant 2013 (607448). Further development of the eSALT program was partially supported by a University of Queensland Early Career Researcher Grant 2015 (609878). This research was conducted with the support of the Centre of Research Excellence in Telehealth funded by the National Health and Medical Research Council (NHMRC; grant ID: APP1061183)

### Conflict of interest statement

The authors AH and HB are domestic partners and so a potential perceived conflict of interest arose when AH received funding through a UQ Early Career Research Grant for $ 35,905.61 of which $ 8,318.98 was budgeted to pay HB for 120 h of casual work to further refine the software program eSALT. HB was employed to make these refinements as eSALT is an implementation of HB's software program AppTailor. All perceived conflicts of interest surrounding this research have been declared and lodged with the Research and Innovation Division of The University of Queensland. eSALT is not a commercial product.
